# Manipulation of film quality and magnetic properties of CrO_2_ (100) films on TiO_2_ substrates with carrier gas and growth temperature

**DOI:** 10.1039/c7ra10874e

**Published:** 2018-01-05

**Authors:** Ming Cheng, Zhihong Lu, Zhenhua Zhang, Ziyang Yu, Shuo Liu, Changwei Chen, Yuting Li, Yong Liu, Jin Shi, Rui Xiong

**Affiliations:** The State Key Laboratory of Refractories and Metallurgy, College of Science, Wuhan University of Science and Technology Wuhan 430065 People's Republic of China zludavid@live.com; School of Materials and Metallurgy, Wuhan University of Science and Technology Wuhan 430081 People's Republic of China; Key Laboratory of Artificial Micro- and Nano-structures of Ministry of Education, School of Physics and Technology, Wuhan University Wuhan 430072 People's Republic of China xiongrui@whu.edu.cn

## Abstract

High-quality CrO_2_ films were synthesized on TiO_2_ (100) substrates at different temperatures using the chemical vapor deposition method in argon or nitrogen atmosphere. It was found that the lower limit for the growth temperature of CrO_2_ films can be reduced to 310 or 300 °C when using Ar or N_2_ as the carrier gas, respectively. The quality of CrO_2_ film on TiO_2_ substrate can thus be improved by optimizing growth temperature in a much larger range (310–400 °C in Ar and 300–430 °C in N_2_, in contrast with 390–410 °C in O_2_), which is significant for the practical application of CrO_2_ films. The best film quality was achieved at 320 °C in either Ar or N_2_ atmosphere, at which CrO_2_ film has its narrowest orientation distribution and lowest roughness. Compared to films grown in O_2_, films grown in Ar were found to have larger saturation magnetizations (*M*_s_) and magnetic anisotropies, possibly due to numerous O vacancies. Films grown in N_2_ are actually N-doped films, and have lower *M*_s_ than those grown in O_2_. The Curie temperature (*T*_c_) was also tuned by the carrier gas and growth temperature. Films grown in Ar or N_2_ generally have a higher *T*_c_ value than those grown in O_2_. Furthermore, the thermal stability of the films was found to be remarkably improved when using N_2_ as the carrier gas.

## Abstract 2

The quality and properties of CrO_2_ films can be manipulated by changing the carrier gas and varying the deposition temperature.

## Graphical abstract

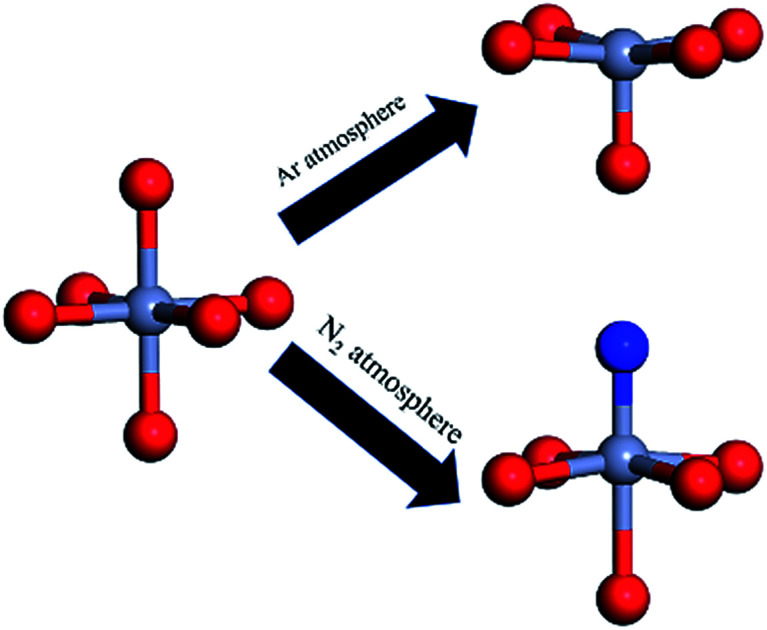

## 1. Introduction

Since chromium dioxide (CrO_2_) was first theoretically predicted as a kind of half metal (HM) ferromagnet by Groot in 1983,^1^ its half metallicity has been confirmed by different experimental technologies, such as Meservey–Tedrow spin-polarized tunneling^2^ and point-contact Andreev reflection.^3,4^ As a HM material, CrO_2_ only has one spin channel involved in electron transport due to its special band structure, which makes the charge carriers 100% spin polarized.^5–7^ This distinctive property of CrO_2_ makes it very promising in the synthesis of high performance spintronic devices, such as giant magnetoresistant (GMR) devices, magnetic tunnel junctions (MTJ), and magnetic random-access memory (MRAM).^8–11^ Although much effort has been devoted in past years, CrO_2_ has never been successfully utilized in spintronic devices. One primary obstacle for the practical application of CrO_2_ concerns the difficulties for obtaining high films quality due to other metastability and stringent preparation conditions of CrO_2_ films. Chemical vapor deposition (CVD) in oxygen atmosphere is a commonly used method to synthesize epitaxial CrO_2_ films.^12–14^ As a metastable state material, CrO_2_ easily decomposes into Cr_2_O_3_ which is an antiferromagnetic and insulator in room temperature under air atmosphere.^15,16^ To prevent the degradation of CrO_2_, isostructural rutile TiO_2_ substrates are usually used. However, on TiO_2_ substrates, CrO_2_ films can only be synthesized in a narrow temperature range of 390–410 °C in an oxygen rich atmosphere. Below 390 °C, CrO_2_ films cannot form due to the lack of interfacial energy needed for bonding or nucleating on the surface of TiO_2_. Above 410 °C, detectable Cr_2_O_3_ phase will appear in films due to thermal instability.^13^ This narrow temperature window greatly limits the qualities of CrO_2_ films for two prominent reasons. Firstly, the growth temperature is so close to the upper limit at which observable film degradation happens that the existence of tiny Cr_2_O_3_ in films may be unavoidable. Secondly, there is no space for improvements in film quality through temperature optimization. Considering device fabrication, low temperature growth is desirable to decrease the interface diffusion and simplify the fabrication process. Therefore, for the practical application, it is important to explore a method to expand the temperature window and grow CrO_2_ films at a lower temperature. Recently, Sousa^17^*et al.* successfully fabricated CrO_2_ films on Al_2_O_3_ substrates at a temperature as low as 330 °C, broadening the process window by 50 °C. However, Al_2_O_3_ may be unable to play the same role as TiO_2_ in stabilizing CrO_2_ film. Considering that Al_2_O_3_ substrates have the same hexagonal structure as Cr_2_O_3_, and the lattice constant differences between them are less than 5%, Cr_2_O_3_ may easily form at the interface of CrO_2_/Al_2_O_3_. This kind of interface degradation was observed in experiments.^18^ To broaden the temperature window for the growth of CrO_2_ and avoid the interface degradation, it is highly significant to lower the growth temperature of CrO_2_ films on TiO_2_ substrates.

According to binary alloy phase diagrams,^19^ researchers have proposed a common theorem that CrO_2_ epitaxial films can only be manufactured under enough high oxygen pressure, where O_2_ is usually used as the carrier gas to avoid the formation of Cr_2_O_3_.^20^ Most recently, Duarte^21^*et al.* obtained (110) oriented pure CrO_2_ films on TiO_2_ substrates using argon as a carrier gas, suggesting that an oxygen rich atmosphere may not be necessary for the growth of purely-phase CrO_2_ films. However, in the Duarte's study, pure (100) oriented CrO_2_ films were not obtained, and the growth temperature was not decreased.

In the presented work, (100) CrO_2_ films on TiO_2_ substrates were fabricated at different temperatures using argon or nitrogen as the carrier gas. It was found that high quality CrO_2_ films can be obtained at a temperature as low as 310 and 300 °C using Ar or N_2_ as the carrier gas, respectively. And the process the temperature windows are 310–400 °C and 300–430 °C, respectively, under Ar or N_2_ atmosphere. The surface morphology, orientation distribution, and magnetic properties of the films fabricated at different temperatures and under different atmospheres were investigated and subsequently discussed.

## 2. Experimental details

A two zone CVD furnace at atmospheric pressure was utilized to synthesize the CrO_2_ films, since CVD is the best way to manufacture CrO_2_ epitaxial films as suggested by previous studies.^20^ CrO_3_ powder (purity 99.9%) was placed in low temperature zone as the chromium source and heated to 260 °C. 5 × 5 mm single crystal rutile TiO_2_ substrates with the direction of one in-plane crystal axis marked were introduced to the high temperature zone at various temperature. Oxygen (purity 99.99%), argon (purity 99.99%), and nitrogen (purity 99.99%) were used as the carrier gases, and the flow rate was fixed to 160 sccm. The quality of the CrO_2_ films was claimed to be critically dependent on the substrate temperature, so to investigate the effects of growth temperature on film quality, films were grown at different temperatures in the range of 300–450 °C. Before film deposition, the substrate was pretreated with hydrofluoric acid (HF) in an ultrasonic dispersion cleaner for 5 min, and then cleaned with acetone for another 5 min. In order to prevent the deposition of undesirable compounds during the initial stage of the deposition process, the substrate was heated to the deposition temperature before the CrO_3_ precursor reached its melting temperature (196 °C). The thickness of all the samples used in this study are around 100 nm.

The crystallographic structure and phase of the deposited films were investigated by X-ray diffraction (XRD) using a Bruker D8 X-ray diffractometer with Cu K_α_ radiation. The thickness of the films were evaluated using scanning electron microscopy (SEM) from the cross-section images, while the surface morphology and roughness were characterized using atomic force microscopy (AFM). The elemental compositions of the films were determined by X-ray photoelectron spectroscopy (XPS). The magnetic properties of the films were studied using a vibrating sample magnetometer (VSM) at room temperature with an applied magnetic field parallel to *c*- or *b*-axis.

## 3. Results and discussion

### 3.1 Phase and structure

**Fig. 1:**
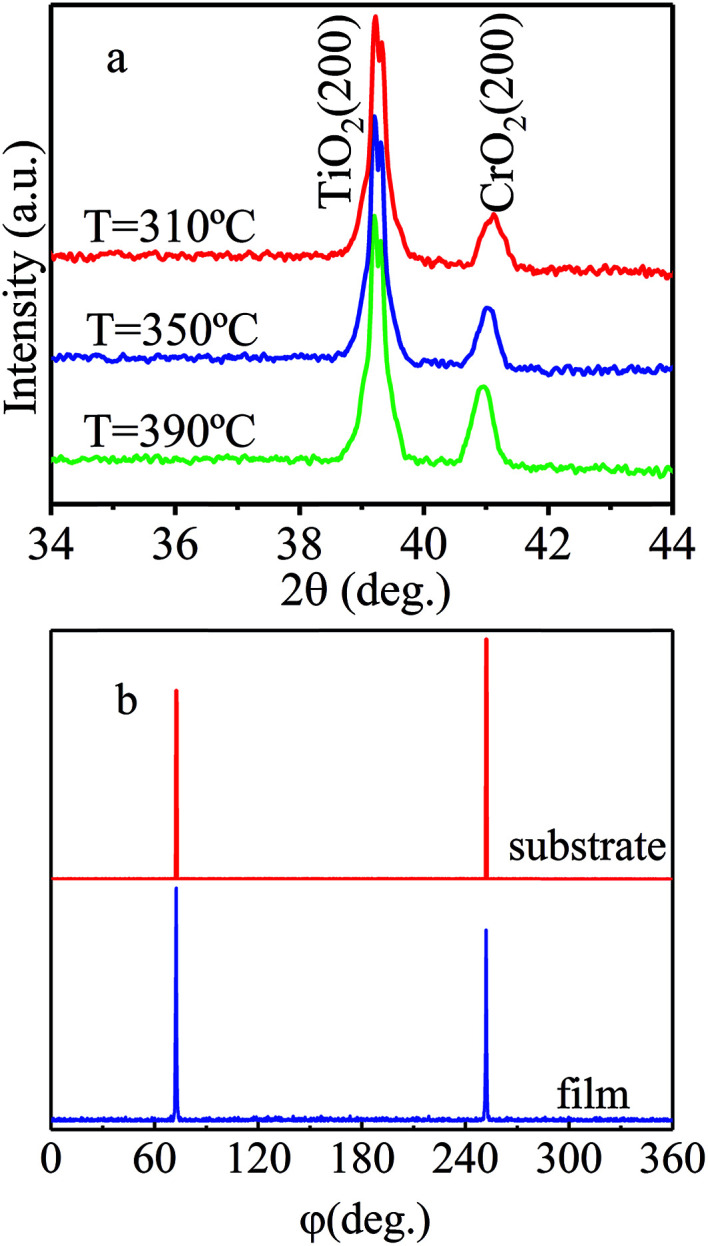
(a) The XRD patterns of films synthesized at different temperatures when argon was used as the carrier gas. (b) {110} phi-scan of CrO_2_ film with the film rotating to the (110) plane under argon atmosphere.

To investigate the phase and structure of CrO_2_ films grown at different temperatures in Ar atmosphere, XRD measurements were performed in the scan angle range of 10–90°. XRD results suggest that pure phase (100) CrO_2_ films can be obtained in a growth temperature range of 310–400 °C. Fig. 1(a) shows the XRD patterns (for better observation, the value of *y*-axis is taken logarithm) for (100) oriented CrO_2_ films fabricated at different temperatures when the carrier gas was argon. Here, we only show the spectra in the angle range of 34–44°. It is obvious that only the (200) diffraction peaks of the TiO_2_ substrates and CrO_2_ films appear in the spectra, suggesting the films are pure in phase. The epitaxy of the films was examined by performing phi-scan after rotating the horizontal plane from the (100) plane to (110) plane. Here, we use the film grown at 310 °C as an example and show its {110} phi-scan in Fig. 1(b). For comparison, the {110} phi-scan of TiO_2_ substrate is also shown. The appearance of (110) and (1̄10) peaks suggest that the CrO_2_ film and the TiO_2_ substrate have the same two-fold rotational symmetry along the [110] axis. The corresponding peaks of the film and substrate locate exactly at the same angle, revealing that the CrO_2_ film is epitaxially grown on the TiO_2_ substrate.

Based on above results, pure CrO_2_ (100) film can be grown at a much lower temperature in Ar atmosphere than film in O_2_ atmosphere. However, it is also noticed that the upper limit of temperature is 400 °C, which is 10 °C lower than that in O_2_ atmosphere.^13^ The reduction of growth temperature upper limit indicates that films fabricated in Ar atmosphere may be less stable than those prepared in O_2_ atmosphere.

**Fig. 2:**
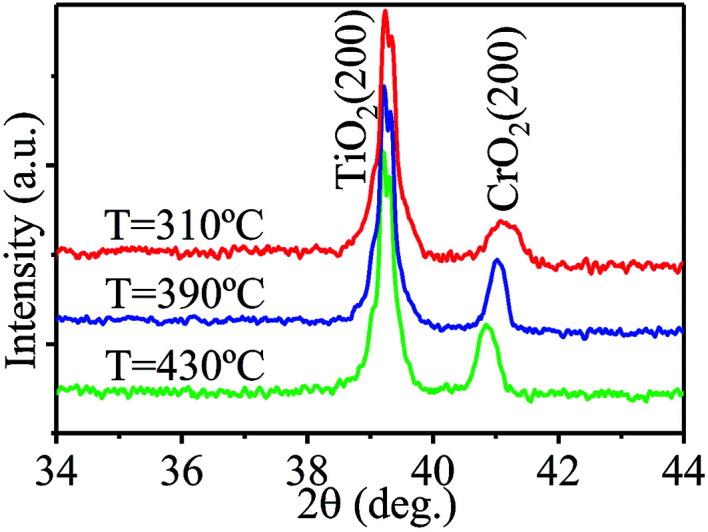
The XRD patterns of films synthesized at different temperatures when nitrogen was used as the carrier gas.

When nitrogen was used as the carrier gas, purely phased epitaxial CrO_2_ films were also obtained. Fig. 2 shows XRD spectra of CrO_2_ films grown in nitrogen atmosphere at different temperatures. All the spectra only show the (200) peaks of CrO_2_ and TiO_2_, while no other phases are detectable in the films. The epitaxy of the films was also confirmed by a {110} phi-scan (not showed). Although the lowest temperature shown in Fig. 2 is 310 °C, according to our study, purely phased CrO_2_ can be obtained at a temperature as low as 300 °C when N_2_ is used as the carrier gas. Therefore, the temperature window for film growth is 300 to 430 °C in N_2_ atmosphere, which is broadened by about 110 °C relative to that in oxygen atmosphere. Compared to the temperature window for film growth in argon atmosphere, CrO_2_ film can be grown at an even lower temperature in nitrogen atmosphere. Another important feature to note is that, purely phased CrO_2_ films can be obtained at a temperature as high as 430 °C in N_2_ atmosphere—which is 20 and 30 °C higher than in O_2_ and Ar atmosphere, respectively. The enhancement of upper limit of growth temperature implies that films prepared under nitrogen atmosphere may have better stability than those grown under O_2_ or Ar atmosphere.

In O_2_ atmosphere, CrO_2_ films cannot be synthesized below 390 °C due to the lack of interfacial energy needed for bonding or nucleating on the surface of TiO_2_.^22^ However, in Ar or N_2_ atmospheres, CrO_2_ films can be obtained at much lower temperatures, indicating that N_2_ and Ar may help to lower the energy barrier for film bonding or nucleating. However, this kind of role performed by N_2_ or Ar may be surface selective. It was found that CrO_2_ films can only be prepared on the TiO_2_ (110) substrates at a temperature equal to or higher than 380 °C in N_2_ or Ar atmosphere.

### 3.2 Film quality

To investigate the effects of growth temperature on film quality, the surface morphologies and orientation distributions of CrO_2_ films grown at different temperatures were studied.

**Fig. 3:**
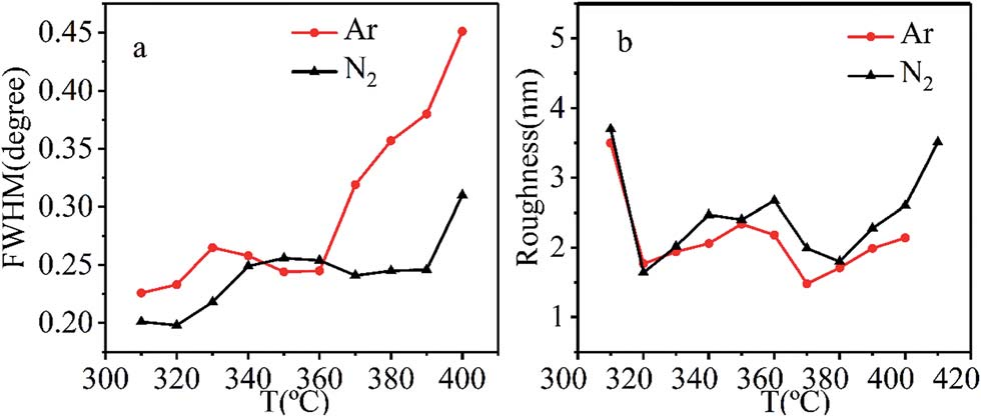
(a) The FWHM of rocking curve for CrO_2_ (200) peak as a function of growth temperature and (b) the roughness as a function of growth temperature with different carrier gases.

The orientation distributions of grains in films were evaluated by analyzing the full width at half maximum (FWHM) of the rocking curve. The FWHMs of films synthesized at different temperatures using Ar or N_2_ as the carrier gas are shown in Fig. 3(a). In oxygen atmosphere, the lowest FWHM of 0.27° is obtained for films at 390 °C, which indicates the harsh synthesis condition needed to synthesize high-quality CrO_2_ films in oxygen atmosphere. According to Fig. 3(a), FWHMs lower than this value can be achieved in a large temperature range in Ar or N_2_ atmosphere, where a FWHM lower than 0.27° could be obtained in a temperature range of 310–360 °C. A FWHM lower than 0.27° could be obtained in a temperature range of 310–390 °C in N_2_ atmosphere. Generally speaking, low FWHMs are usually obtained at low temperatures. As the temperature increases, FWHM also increases significantly. In Ar atmosphere, the lowest FWHM is 0.22° and obtained at 310 °C, while in N_2_, the lowest FWHM is 0.19° and obtained at 320 °C. Both of these FWHM values are significantly lower than that obtained in O_2_ which is 0.27°. The results suggest that the decrease in growth temperature using Ar or N_2_ as the carrier gas is beneficial for improving the quality of CrO_2_ films. Comparatively, at most temperatures, films grown in N_2_ atmosphere have lower FWHMs than their counterparts fabricated in Ar atmosphere.

The roughness of the films was evaluated using AFM and is depicted in Fig. 3(b). Films grown in Ar atmosphere have roughness around 2 nm. The lowest roughness appears in film grown at 370 °C. For films grown in N_2_ atmosphere, the roughness fluctuated from 1.3 to 2.5 nm in temperature range of 320–400 °C. As the temperature increased beyond 400 °C, or decreased below 310 °C, the roughness became larger than 3.5 nm.

Considering both orientation distribution and roughness, the best fabrication temperature was determined to be 320 °C for either Ar or N_2_ atmosphere.

**Fig. 4:**
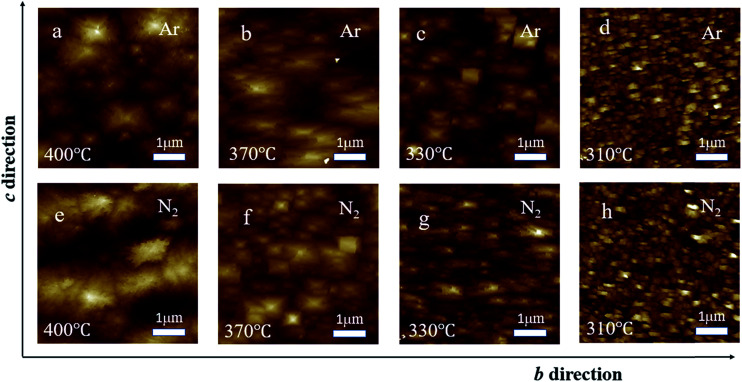
The AFM image of films synthesized under Ar atmosphere when the temperature was set to (a) 400, (b) 370, (c) 330, and (d) 310 °C; the AFM image of films synthesized under N_2_ atmosphere when the temperature was (e) 400, (f) 370, (g) 330, and (h) 310 °C.

To investigate the surface morphology, AFM images for films synthesized at 400, 370, 330, and 310 °C are shown in Fig. 4. The surface morphology seems to depend on both the growth temperature and the type of the carrier gas. At 310 °C, the film surface was covered by small grains with spiny shapes for both Ar and N_2_ cases. As the growth temperature increased to 330 °C, the surface of the film grown in Ar atmosphere is composed of platelet-like grains with square shape, while the surface of films grown in N_2_ atmosphere exhibited enlarged grains with the length direction along the *b*-axis. At high temperatures (from 370 to 400 °C), the morphology of the films grown in Ar or N_2_ is of nodular type consisting of particles composed of numerous large or small grains. However, the particles in films grown in different atmospheres possessed different shapes. The particles in the film grown in Ar are random, while those in the film grown in N_2_ exhibit a rectangular shape with the long sides along the *b*-axis. The types of surface morphology at different temperatures and under different carrier gases may be the reason for the temperature and carrier gas dependent roughness.

### 3.3 Magnetic properties

**Fig. 5:**
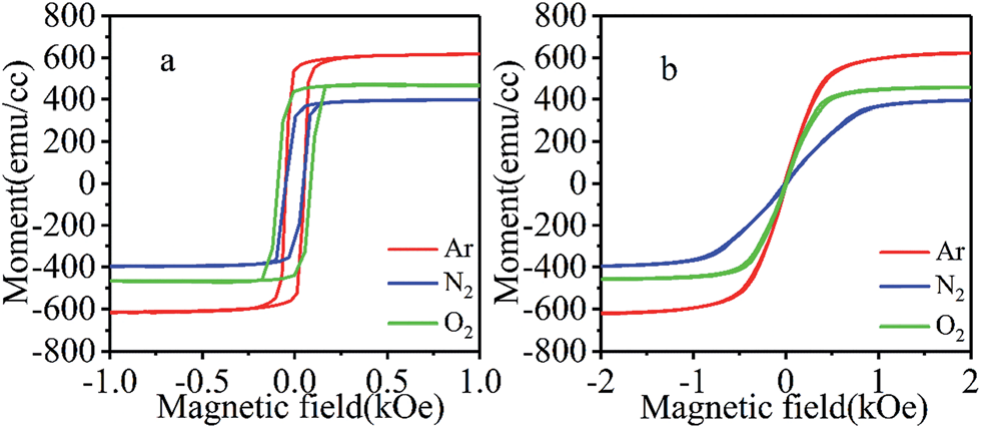
The hysteresis loops of films synthesized under three different atmospheres when the field is along (a) the [001] or (b) [010] direction.

To investigate the magnetic properties of CrO_2_ films, hysteresis loops were measured at room temperature (300 K) with an in-plane magnetic field applied along the [010] or [001] direction. Here, we put the hysteresis loops of films grown at same temperature but in different atmospheres in the same figure to compare their magnetic properties. Fig. 5(a) and (b) show the easy and hard axis loops of samples deposited at 390 °C under argon, nitrogen, and oxygen atmosphere, which reveals that all films have good uniaxial magnetic anisotropy. Despite being grown under different carrier gases, the easy axes of the samples are along the *c*-axis, while the hard axes are along the *b*-axis. Results also indicate that the film fabricated in Ar has a larger, and that grown in N_2_ has a lower saturation magnetization (*M*_s_) than the one deposited in O_2_. According to the hard axis loops shown in Fig. 5(b), films grown in different atmospheres have different switching fields (*H*_k_), suggesting that their magnetic anisotropies may also vary.

To comprehensively investigate the effects of growth temperature and atmosphere, the magnetic properties of films synthesized at different temperatures and in different atmospheres are summarized and listed in Table 1. Compared with *M*_s_ value of 460 emu cm^−3^ (for the film grown in oxygen atmosphere), *M*_s_ value of the films grown in argon atmosphere are much higher, while *M*_s_ values of those grown in N_2_ are significantly lower. In addition to the dependence on growth atmosphere, *M*_s_ also shows dependence on growth temperature. As the growth temperature changes, *M*_s_ also varies to some extent. Considering that Ar is an inert gas, it will not take part in chemical reactions for film formation when being used as a carrier gas. However, the existence of a large amount of Ar around the substrate may lead to a large number of oxygen vacancies in the obtained films due to O deficiency. To confirm this, the elemental compositions of the surfaces of the film grown in Ar were analyzed by XPS (results not shown) and compared with those synthesized in O_2_. It was found that the *N*_O_/N_Cr_ ratios (*N*_O_ and *N*_Cr_ refer to the numbers of O atoms and Cr atoms, respectively) of the films grown in Ar are much lower than those of the films in O_2_, indicating that more O vacancies may exist in them, at least on the surface. In defect-free CrO_2_, each Cr atom loses 4 electrons to six neighbored O atoms and becomes Cr^4+^ ion. The two left 3d electrons will be filled in majority t_2g_ states. Therefore, each Cr ion possesses around 2 *μ*_B_ moment. When one O vacancy appears around a Cr ion, the Cr ion will lose fewer electrons. Although appearance of the O vacancy will lead to small changes in the energies of 3d states of the Cr due to the variation of crystal field, majority t_2g_ states still have lower energies than minority ones. All 3d ion electrons of the Cr will be filled in majority states. As a result, the moment of the Cr ion will increase^23^. When a Cr ion loses more than one neighbored O atoms, its minority 3d states may have lower energy than some majority ones due to large crystal field splitting and be partially filled, which may lead to a decrease of the moment of the Cr ion. However, in this case, the moments of Cr ions in neighbored octahedrons may increase significantly since each O vacancy is shared by three Cr ions (read reference [23] for detail). As a result, the total moment still increases. In the discussion above, we suppose the density of O vacancy in CrO_2_ is not that high and there no vacancy aggregation. This supposition may be reasonable because very high density and aggregation of O vacancy may lead to the appearance of Cr_2_O_3_ phase. In our films, no Cr_2_O_3_ was detected. Based on the discuss above, the larger *M*_s_ of films fabricated in Ar atmosphere may be due to existence of a larger number of O vacancies compared to the films grown in O_2_. The variation in the quantity of O vacancies in films grown at different temperatures may the reason for the growth temperature dependence of *M*_s_.

**Table 1:** The coercivities (*H*_c_), magnetic moments (*M*_s_), anisotropy constants (K), and the Curie temperatures of the films synthesized with different carrier gases and at different growth temperature range of 330–390 °C

	*T* (°C)	*H*_c_ (Oe)	*M*_s_ (emu cm^−3^)	*K* (10^5^ erg cm^−3^)	*T*_c_ (K)
Ar	330	80	581.6	1.888	389
	350	51	593.7	1.558	405
	370	77	554.0	1.859	425
	390	74	602.9	2.138	399
N_2_	330	95	425.2	1.221	385
	350	57	392.7	1.397	388
	370	44	431.7	1.276	410
	390	57	400.2	1.508	414
O_2_	390	117	460.0	1.112	387

The analysis of the elemental compositions found that a significant number of N ions exist in the films grown in N_2_; suggesting that they are actually N-doped CrO_2_ films. This finding indicates that N is involved in the chemical reaction for film formation due to its relatively high chemical reactivity. In their first principle study,^24^ Y. Xie *et al.* found N doping reduces the magnetization of CrO_2_. The comparatively lower *M*_s_ of the films grown in N_2_ as shown in Table 1 may be attributed to the substitutions of N atoms for some of the O atoms in the films. To investigate the reasons for the reduction of *M*_s_, density of states of N-doped CrO_2_ was studied using first principle method (results are not shown). It was found, even though the introduction of a N atom slightly changes the energies of 3d states of neighbored Cr ions, the majority t_2g_ states still have lower energy than minority ones. So, the 3d electrons are filled in spin up states. Therefore, the half metallicity of CrO_2_ maintains. However, the crystal field splitting led by N dopants makes the 3d electrons of Cr ions more delocalized, leading to a decrease of the moments of those Cr ions. Moreover, due to their incompletely occupied 2p states, N ions possess larger negative moments than O ions. The reduction of moments of Cr ions and fairly large negative moments of N are the reasons for the decrease of *M*_s_ in N-doped CrO_2_ films. Based on XPS, N concentrations in films grown at 320, 350, 370 and 390 °C were determined to be 2.07%, 2.48%, 3.91%, and 2.39%, respectively. These results show some extent of temperature dependence, which may help to explain the observed growth temperature dependence of *M*_s_.

In coherent switching model, the effective magnetic anisotropy (K) can be calculated using following equation:
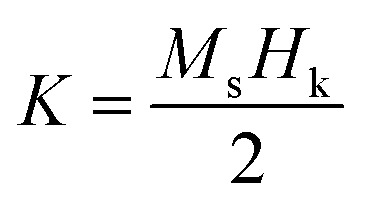
where *M*_s_ and *H*_k_ are the saturation magnetization and the hard axis switching field, respectively.^25^ The anisotropy constants shown in Table 1 indicate that films grown in Ar or N_2_ have higher magnetic anisotropies than those grown in O_2_. The first principle study also suggests that N doping could increase the magnetic crystalline anisotropy of CrO_2_ (results not shown). Therefore, higher anisotropies obtained for films grown in Ar or N_2_ may be attributed to the increase in the quantity of O vacancies or the substitution of N atoms for some of the O atoms in the films. Materials with strong magnetic anisotropy are desirable for the improvement of thermal stability of spintronic devices.^26^ CrO_2_ films with enhanced magnetic anisotropy may have important applications in devices.

The temperature dependence of magnetizations for the films grown at different temperatures and in different atmospheres was measured in a temperature range of 300–500 K with a 500 Oe magnetic field applied along the *c*-axis. The Curie temperatures (*T*_c_) were obtained from the *M*_s_*vs. T* curves, which are shown in Table 1. Generally speaking, the films grown in Ar and N_2_ have higher *T*_c_ value than those grown in O_2_, suggesting that ferromagnetic (FM) phase of CrO_2_ has better stability when synthesized in Ar or N_2_.

### 3.4 Thermal stability

**Fig. 6:**
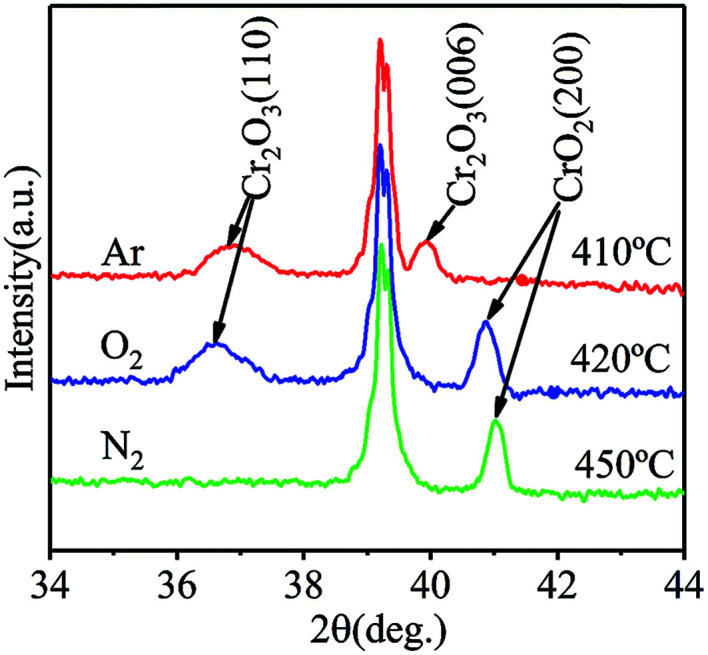
The XRD pattern of CrO_2_ films synthesized (red) in argon atmosphere annealed at 410 °C, (blue) in oxygen atmosphere annealed at 420 °C, and (green) in nitrogen atmosphere annealed at 450 °C.

To evaluate the stability of films fabricated at low temperatures, films grown in Ar or N_2_ at 320 °C were annealed at different temperatures in air for 60 min. The phase and crystal structures of each annealed films were characterized using XRD, and the highest annealing temperature the film can withstand was obtained. The XRD spectra of the films annealed at different temperatures are shown in Fig. 6. After annealing at 410 °C, only diffraction peaks of Cr_2_O_3_ are observable, revealing that CrO_2_ completely degraded into Cr_2_O_3_ in the film grown in Ar. The XRD spectrum without detectable Cr_2_O_3_ peaks is only obtained when the annealing temperature is lower than 400 °C. For films grown in O_2_, significant amounts of Cr_2_O_3_ appears when the annealing temperature reaches 420 °C. Although the main phase is still CrO_2_, the highest temperature it can withstand is 410 °C. For the film grown in N_2_ atmosphere, even after being annealed at 450 °C, no Cr_2_O_3_ peak is detectable in the XRD spectrum. Cr_2_O_3_ phase appears in films grown in N_2_ when the annealing temperature is higher than 470 °C. According to the different tolerances to annealing exhibited by the films, films grown in N_2_ possessed the best thermal stability, while those grown in Ar are least stable. As discussed above, more O vacancies may form in films when Ar is used as the carrier gas in instead of O_2_ due to O deficiency. According to our theoretical study,^23^ once an O vacancy exits in an octahedral of CrO_2_ crystal, new O vacancy tends to form in the same octahedral. As a result, Cr ion in the center of the octahedral will be reduced to Cr^3+^. Therefore, the existence of a larger number of O vacancies in the films grown in Ar make them less stability compared to those grown in O_2_ because Cr_2_O_3_ more easily forms. In the film growns in N_2_, a small number of N atoms substituted for O atoms. Since N is in 2p^3^ valence state and the most stable ionic form of N is N^3−^, a N atom can accept one more electron than an O atom. When N ions exist in a film, they can weaken the effects of O vacancies around them. Therefore, the existence of N ions may retard the reduction of neighbored Cr ions, leading to an enhancement of thermal stability of the film. It is pertinent to mention that N doping does not affect the half metallicity of CrO_2_ according to the results of our first principle study (results are not shown and will be published elsewhere).

## 4. Conclusion

Using Ar or N_2_ as the carrier gas, epitaxial CrO_2_ (100) oriented films were synthesized on TiO_2_ at different growth temperatures. The quality, magnetic properties, and thermal stability of the films were evaluated. The conclusions are summarized as follows:

(1) High quality, pure rutile phased CrO_2_ (100) oriented film can be grown at a temperature as low as 310 °C on TiO_2_ substrate using Ar or N_2_ as the carrier gas.

(2) In Ar and N_2_ atmospheres, the temperature windows for film fabrication were 310–400 °C and 300–430 °C, respectively, which was more greatly broadened that the window in O_2_. This makes it possible to improve film quality by growth temperature optimization.

(3) In Ar and N_2_ atmospheres, films with the best quality were obtained at 320 °C, which both had a narrow orientation distribution and low roughness.

(4) The saturation magnetization, anisotropic energy, and Curie temperature of CrO_2_ films can be manipulated by adjusting the growth temperature and changing the carrier gas.

(5) The thermal stability of CrO_2_ film can be enhanced by using N_2_ as the carrier gas, which may be of great significance for practical applications.

## Conflicts of interest

There are no conflicts of interest to declare.

## Supplementary Material
